# Does clozapine treat antipsychotic-induced behavioural supersensitivity through glutamate modulation within the striatum?

**DOI:** 10.1038/s41380-023-02026-x

**Published:** 2023-03-17

**Authors:** Prashant Tibrewal, Pramod C. Nair, Karen J. Gregory, Christopher J. Langmead, Sherry Kit Wa Chan, Tarun Bastiampillai

**Affiliations:** 1 Cramond Clinic, The Queen Elizabeth Hospital, Woodville South, SA 5011 Australia; 2 Discipline of Psychiatry, University of Adelaide, Adelaide, SA Australia; 3 Discipline of Clinical Pharmacology, Flinders Health and Medical Research Institute (FHMRI) College of Medicine and Public Health, Flinders University, Adelaide, SA Australia; 4 South Australian Health and Medical Research Institute (SAHMRI), University of Adelaide, Adelaide, SA Australia; 5 Drug Discovery Biology and ARC Centre for Cryo-electron Microscopy of Membrane Proteins, Monash Institute of Pharmaceutical Sciences, Monash University, 381 Royal Parade, Parkville, VIC 3052 Australia; 6 Department of Psychiatry, The University of Hong Kong, Hong Kong SAR, China; 7 The State Key Laboratory of Brain and Cognitive Sciences, The University of Hong Kong, Hong Kong SAR, China; 8 Discipline of Psychiatry, College of Medicine and Public Health, Flinders University, Adelaide, SA Australia; 9 Department of Psychiatry, Monash University, Wellington Road, Clayton, 3800 Australia

**Keywords:** Molecular biology, Neuroscience

## Abstract

Behavioural supersensitivity may be a result of increased glutamate sensitivity of D2-MSN and reduced sensitivity to dopamine. We propose that clozapine may address behavioural supersensitivity by modulating glutamate activity which may partially explain its unique effectiveness in the setting of treatment resistant schizophrenia.

The mainstay of schizophrenia treatment is chronic antipsychotic medication to prevent relapse of a psychotic episode. These antidopaminergic drugs reduce D2 dopamine receptor-mediated transmission. It has been proposed that relapse rates following antipsychotic discontinuation may be due to withdrawal phenomenon rather than due to illness recurrence [1]. The leading hypothesis is chronic D2-receptor blockade produces compensatory changes leading to a state of dopamine supersensitivity (or behavioural supersensitivity) by potentiating D2-receptor density/function specifically in the striatum [2]. An alternate hypothesis is that increased glutamate but decreased dopamine sensitivity occurs during chronic antipsychotic treatment implying the role of the glutamatergic system [3]. There is also some scepticism on the existence of dopamine supersensitivity based on strict clinical observation [4].

Kruyer et al. studied the mechanism of antipsychotic-induced behavioural supersensitivity and did not find any change in D2-receptor expression or function in mice administered chronic haloperidol, in the ventral striatum, dorsal striatum or midbrain and concluded neither D2-receptor expression nor the hypothesized increased function of the receptor could explain behavioral supersensitivity [5]. Instead, enhanced glutamatergic transmission on D2-medium spiny neuron during haloperidol discontinuation was observed, accompanied by retraction of astrocytic processes giving rise to excitatory postsynaptic plasticity [5]. Kruyer et al. also demonstrated that by silencing hyperactive D2-medium spiny neuron through restoration of G_i_ intracellular signalling, behavioural supersensitivity was reversed [5].

Based on the above, Kruyer et al. propose D2-medium spiny neurons hyperexcitation in the nucleus accumbens core underpins neuropathology during chronic antipsychotic treatment, antipsychotic discontinuation and behavioural supersensitivity, such that chronic antipsychotic treatment possibly reduces modulatory dopaminergic input making D2-medium spiny neurons hyperexcitable to incoming excitatory transmission from glutamate [5]. Behavioural supersensitivity may therefore be a result of increased glutamate sensitivity of D2-medium spiny neurons and reduced sensitivity to dopamine.

Antipsychotic-induced behavioural supersensitivity is postulated to reduce the efficacy of antipsychotic medication leading to increasing antipsychotic doses, tolerance and treatment resistance. It has also been possibly linked to the development of tardive dyskinesia which is a motor disorder known to be caused by prolonged antipsychotic exposure [2]. A recent meta-analysis by Siskind et al., identified that 25% of patients with first-episode schizophrenia develop treatment resistance within the early stages of the illness [6]. Therefore, treatment resistance in this cohort is unlikely to be due to anitipsychotic-induced behavioural supersensitivity as antipsychotic treatment was already ineffective before producing any neurobiological changes underlying behavioural supersensitivity. However, Siskind et al. identified a further 8% of patients who despite having achieved an initial response to antipsychotics subsequently developed treatment resistant schizophrenia [6] and in this latter smaller cohort dopamine supersensitivity psychosis could be a potential explanatory model. It is also important to note that the majority (two thirds) of the follow-up studies included in this meta-analysis had follow-up periods of less than 5 years and therefore may have significantly underestimated the actual rates of later-onset treatment resistant schizophrenia [6]. Around 20–30% of patients prescribed long-acting injectable antipsychotics will later develop breakthrough psychosis despite having achieved an initial treatment response [7]. A recent 12-year follow-up study of first episode schizophrenia identified only 10% of all treatment resistant schizophrenia cases occurred at illness onset, but by the third episode of illness (second relapse) 61% of patients had developed treatment resistant schizophrenia [8]. Higher antipsychotic doses prescribed within the first 2 years were associated with treatment resistant schizophrenia. Treatment resistant schizophrenia cases also had a continuous increase in daily defined antipsychotic dose within the first 2 years, compared with a stable daily defined dose for non-treatment resistant schizophrenia cases [8]. These findings suggest the possibility of tolerance to antipsychotic medication in treatment resistant schizophrenia patients [8].

Clozapine is a unique medication acting upon multiple receptors [9], and is effective in approximately 40% of treatment resistant schizophrenia patients [10] who do not respond to first-line antipsychotic treatment (D2 antagonists), implying possible explanatory mechanisms beyond dopamine receptors. Clozapine is also likely more effective in tardive dyskinesia in patients with schizophrenia compared to other antipsychotic medication [11–13]. The neurobiological mechanism of clozapine’s effectiveness in these conditions is unclear.

It has been hypothesised that clozapine may specifically modulate brain glutamate and that this effect could contribute to its unique efficacy for patients with treatment-resistant schizophrenia [14, 15]. Glutamate through its actions at ionotropic and/or metabotropic receptors has multiple brain functions including neural plasticity, neural network formation and central nervous system repair [16]. Excessive levels of glutamate are associated with excitotoxicity and neural degeneration [16]. N-methyl-D-aspartate receptor (NMDA-R) is an ionotropic glutamate receptor. Antagonism of NMDA-R by substances like ketamine and phenycyclidine has been shown to replicate the positive, negative and cognitive symptoms of schizophrenia, leading to the NMDA-R hypofunction hypothesis of schizophrenia [16]. Treatment resistant schizophrenia has been associated with higher striatal glutamate and normal dopamine levels [17, 18]. It has been postulated that striatal glutamate levels may continue to remain elevated in patients with treatment resistant schizophrenia and could therefore be one of the targets of action for clozapine [15]. A proton magenetic resonance spectroscopy study comparing glutamate neurometabolites in treatment responsive and resistant schizophrenia patients found that patients with treatment-resistant schizophrenia had higher total glutamate + glutamine levels scaled to creatine in the putamen and considered it as a marker of response to clozapine [19].Clozapine in a randomised double blind controlled trial significantly reduced ketamine-induced psychosis indicating its potential action on NMDA-R [20]. It is hypothesised that clozapine could have specific intrinsic or partial agonist activity at the glycine binding site of NMDA-R ^14^and may also potentiate glutamate transmission by regulating glutamate transport [21]. Tanahashi et al. [22] also demonstrated in a rat model that clozapine but not haloperidol primarily increases glial D-serine and secondarily increases L-Glutamate, which both act as NMDA-R activators. It has also been observed that chronic clozapine rather than haloperidol promotes glutamate release upon potassium stimulation specifically within the nucleus accumbens [23]. Clozapine has been shown to increase C-Fos protein expression a marker of neuronal activity within several brain regions including both the caudate-putamen and nucleus accumbens, but this has not been directly linked to clozapine’s effect on glutamate modulation [24]. In an animal model of schizophrenia (neonatal lesion of the ventral hippocampus) reconstitution of dendritic arbors and synaptogenesis in frontal cortex and nucleus accumbens were observed following clozapine treatment [25] and this was linked to possible interface of clozapine with glutamate [26]. Glutamatergic neurons derived from patients with schizophrenia revealed deficits in electrophysiological properties, synaptic function and network activity [26]. Deficits in network behavior, and glutamatergic synaptic signalling were restored by clozapine but only in glutamatergic neurons from clozapine-responsive patients [26].

However, despite the evidence for glutamatergic dysfunction in schizophrenia, specific pharmacological strategies have so far been largely unsuccessful [16]. These add-on glutamatergic agents focussed on treating negative and cognitive symptoms of schizophrenia, targeting the NMDA-R and have included compounds such as glycine, D-serine, D-cycloserine and D-alanine [16].

There is indirect evidence to support that the efficacy of clozapine in refractory schizophrenia may be related to its modulatory effect on glutamate activity [27–30]. However, most of these studies have examined peripheral glutamate levels and there is paucity of research examining the effect of clozapine on brain glutamate levels and the relationship between peripheral and central glutamate levels [15]. There has only been 1 study conducted, measuring the longitudinal effects of clozapine (before and following clozapine commencement) on glutamate specifically within the caudate [15]. McQueen et al. studied the effect of clozapine on brain glutamate by examining glutamate levels in caudate and anterior cingulate cortex (ACC) in patients with treatment resistant schizophrenia after 12 weeks of commencing clozapine treatment [15]. This study found a reduction in glutamate levels in the caudate which was also correlated with clinical improvement, but no change noted in the ACC [15]. They concluded that the specific reduction in caudate glutamate levels could contribute to the clinical improvements observed on clozapine treatment within the first 12 weeks. We hypothesise based on the above and Kruyer et al.’s findings [5]. that by reducing caudate glutamate levels, clozapine could prevent the development of behavioural supersensitivity and even reverse established behavioural supersensitivity.

The efficacy of clozapine in reversal of tardive dyskinesia can provide indirect links to understand this mechanism further. Antipsychotic-induced behavioral supersensitivity has been considered a possible aetiological factor for the development of both treatment resistant schizophrenia and tardive dyskinesia [2]. Meta-analysis of clozapine treatment for tardive dyskinesia confirms its effectiveness for this difficult to treat condition [31]. Previously it has been hypothesised that clozapine’s rapid dissociation from D2-receptors may explain its potential effectiveness for treatment resistant schizophrenia, supersensitivity psychosis, and tardive dyskinesia [12]. However, quetiapine, which also has rapid D2-receptor dissociation kinetics [32], has not been specifically recommended for treatment resistant schizophrenia within various treatment guidelines [33]. It therefore seems likely that biological mechanisms beyond rapid D2-receptor dissociation may be responsible for clozapine’s demonstrated effectiveness for both treatment resistant schizophrenia and tardive dyskinesia and whether behavioural supersensitivity was the common link. Of note, however, Kruyer et al. did not find a correlation between vacuous chewing movements and behavioural supersensitivity. Further specific research is required to link clozapine, glutamate, and any impact upon tardive dyskinesia.

Kruyer et al., show antipsychotic-induced behavioural supersensitivity may be a consequence of hyperexcitation of D2 medium spiny neuron in the nucleus accumbens core, due to enhanced glutamatergic transmission [5]. Therefore, potential treatments for antipsychotic-induced behavioural supersensitivity may need to have a specific glutamate modulating effect. As discussed above, clozapine’s superior effectiveness for treatment resistant schizophrenia may relate to its ability to address antipsychotic-induced behavioural supersensitivity mediated via potentially modulating glutamate activity, rather than specifically addressing D2-receptor upregulation.

Kruyer et al., have suggested the potential role of deep brain stimulation and transcranial magnetic stimulation, in reducing and preventing excitatory plasticity in D2 medium spiny neuron [5]. However, we hypothesize that clozapine might be the preferred option to potentially treat antipsychotic-induced behavioural supersensitivity and Electro Convulsive Therapy might be the current preferred method of neurostimulation to reverse antipsychotic-induced behavioural supersensitivity ahead of deep brain stimulation and transcranial magnetic stimulation [34].

Understanding the mechanistic basis for antipsychotic-induced behavioural supersensitivity may lead to better prevention efforts (reducing both antipsychotic dose and usage) as well as facilitate targeted treatments for supersensitivity psychosis and tardive dyskinesia. Future research should specifically investigate clozapine’s role in antipsychotic-induced behavioural supersensitivity (animal and human studies) and the mechanism by which clozapine (and other therapeutic approaches) may modulate glutamate-induced excitability specifically within the striatum and the nucleus accumbens. Future controlled trials should be conducted studying the effect of clozapine, electroconvulsive therapy, deep brain stimulation, transcranial magnetic stimulation and other glutamate modulating compounds for the treatment of supersensitivity psychosis with concomitant assessment of glutamate activity within the striatum and nucleus accumbens. These studies may also help identify biomarkers for treatment resistant schizophrenia and possible predictors of clozapine response.

The aetiology of treatment resistant schizophrenia is likely complex and treatments that just target either dopamine or glutamate transmission are less likely to be successful [35]. There is increasing evidence to suggest extensive and complex interactions between the dopamine and glutamate system [35]. It is possible that clozapine may be uniquely effective in treatment resistant schizophrenia and for behavioural supersensitivity because it may modulate dopamine and glutamate simultaneously within relevant brain regions. However further studies are required to understand the role of clozapine on glutamatergic systems at caudate/putamen and the nucleus accumbens.
